# Supplementary material for: Initial growth rates of malware epidemics fail to predict their reach
Source: Sci Rep. 2021 Jun 3;11:11750. doi: 10.1038/s41598-021-91321-0 (PMC8175743; doi:10.1038/s41598-021-91321-0)
Supplement: Supplementary file 1 — Supplementary Methods. [file 41598_2021_91321_MOESM1_ESM.docx]

Supplementary Material for

Initial growth rates of malware epidemics fail to predict their reach

Lev Muchnik, Elad Yom-Tov, Nir Levy, Amir Rubin, Yoram Louzoun

Correspondence to: louzouy@math.biu.ac.il

# Materials and Methods

## Data

Files are identified as malware through several mechanisms. First, customers may flag files as malware and make them known to the antimalware vendor. This happens rarely and mostly for malware causing very severe and noticeable damage. Second, suspicious files might be sent for analysis and identified as malware by the vendor. Finally, other antimalware vendors may share the results of their analysis with the vendor. Once malware is identified, the vendor can decide to block the malware through a signature developed and transmitted to the client machines.

Similar to data from biological viruses and other contact processes, our data may be biased by the behavior of malware vendors and their decision on whether to “vaccinate” machines. To estimate whether vendor policy could influence our results we measured the correlation between the time passed from the earliest observation of the malware hash to its classification as malware and the number of  machines infected within the first 72 hours. The correlation is 0.03, indicating a very weak association between the rise of the malware and the time until a signature will be developed for it. Similarly, the correlation between the absolute number of infected machines after 72 hours and the time between identification and marking was -0.06.

## Methods

### Power law distributions

Power law fits were executed with the python power law toolbox (1) (2). The fits were executed for the integer data (discrete parameter set to true) and with x-minimal boundary set to 1000 for figure 1A and 500 for figure 1C.

### Computing the average hourly growth rate.

The growth rate is averaged over the period of the active spread of each malware, that does not exceed the initial 72 hours. The period of active spread is the time window between the first and the last infection within the first 72 hours of the malware's existence. This definition is chosen to avoid bias to the computed average spread rate from the periods prior to the actual spread of the malware or after its spread had haltered. The average hourly malware growth rate is defined as $\left\langle\frac{dI(t)}{I(t)} \right\rangle$ where $I\left( t \right)$ is the total number of infected at time $t$ and $dI\left( t \right)\equiv I\left( t \right)-I\left( t-1 \right)$. This analysis is executed only for the malwares that had reached 80% of their final reach in the first 72 hours of their spread. Confidence intervals represent standard error (SE).

### SIR Simulation

We test our model by implementing a classical SIR simulation with the following reactions:

- According to the random mixing assumption, each susceptible individual can be infected with a probability proportional to the total number of infected $I(t)$ at time t.
- Each infected can be removed with a constant probability. This probability was varied among simulations.
- The population is seeded at time $t=0$ with $10$ infected individuals chosen randomly.

At each step, the total probabilities of infection or removal are computed, using an efficient sum over all possible events, with an event tree. The event tree leaves are all possible events and the internal nodes are the sum of probabilities in the leaves. Following, the choice between infection or removal, the target of the infection and removal is chosen in the tree.

The simulation is executed over 1,000,000 individuals, each with an individual value $\beta_{i}$ representing her characteristic susceptibility. The values $\beta_{i}$ were drawn from a scale-free distribution $p\left( \beta\right)\sim\beta^{-\alpha}$ with the slopes $\alpha=2.5$ (similar to the observed in the malware infectivity data) and $\alpha=1.5$ – to demonstrate the effect of heterogeneity. The figure in the main article (Fig. 1D) represents the average susceptibility of the infected population as the simulation progresses, computed over 10,000 of the simulation runs.

The code is available as a matlab code in the Supp. Mat.

In the figures, we used values of delta and beta on a log scale between ${10}^{-4}$and ${10}^{-1}$ for delta and ${10}^{-6}$ and ${10}^{-1}$ for beta. For delta the rate is absolute. For beta, the value of beta is the value for the minimal infectivity.

### Malware reach null model

In figure 3A and 3C, we compare the distribution of malware susceptibilities with the expected one in a null model. In this null model, we mixed the relations between malwares and computers. Formally, we had a list of computers and the malwares that infected them, and the time that the infection occurred. We then scrambled the malwares, so that each malware is associated with a random machine. This procedure can be seen as a configuration model applied to a bipartite machine-malware graph. It preserves both the reach of every malware and the number of infections of every machine. Only the tendency of a malware to affect specific machines is removed. We then analysed the machine susceptibility distribution and its relation with the malware reach.

# Supplemental Tables and Figures

| # | rank | number of malwares | mean malware infectivity | median malware infectivity | mean machine susceptibility | mean machine susceptibility (simulated) |
| --- | --- | --- | --- | --- | --- | --- |
| 1 | 1-2 | 2 | 2580082 (891456) | 2580082 | 1.92 (0.0) | 9.15 (0.0) |
| 2 | 3-9 | 7 | 156452 (15332) | 169254 | 1.63 (0.01) | 9.3 (0.01) |
| 3 | 10-19 | 10 | 64425 (4451) | 56999 | 3.81 (0.02) | 9.24 (0.02) |
| 4 | 20-49 | 30 | 31807 (3891) | 26200 | 5.58 (0.02) | 9.25 (0.02) |
| 5 | 50-99 | 50 | 14369 (314) | 14594 | 7.54 (0.02) | 9.16 (0.02) |
| 6 | 100-499 | 400 | 4868 (140) | 3956 | 4.98 (0.02) | 9.22 (0.02) |
| 7 | 500-999 | 500 | 2496 (130) | 2084 | 7.22 (0.04) | 9.24 (0.04) |
| 8 | 1000-39236 | 38237 | 554 (5) | 368 | 13.24 (0.01) | 9.2 (0.01) |
| 9 | 1-39236 | 39236 | 840 (99) | 376 | 9.29 (0.01) | 9.2 (0.01) |

Table S1. The table summarizes statistical properties of the machines infected by malwares grouped into 8 groups by their reach in the first 72 hours of spread. The last row represents all malwares in this set. The table covers malwares that reached over 200 machines during that period. Malwares in each row are grouped by their rank (2nd column). The third column lists the number of malwares in each group and each followed by the mean (4th column) and median (5th column) number of machines affected by them. The mean susceptibility of the machines infected by the malwares in the group is listed in the 6th column. The last column represents the mean susceptibility of the malwares in the simulated spread that preserved the reach of each malware and susceptibility of each machine with the propensity proportional to the number of infections observed on this machine (see the null model specified above). By yielding constant mean machine susceptibility across all malware groups this test demonstrates that the increase in the mean machine susceptibility with the drop in the malware reach (column six) is not a statistical artifact, but a direct result from selective targeting of malwares. See Figure S2 for details of the distribution of susceptibility for each set of simulations and Fig 3B and Fig 3C for graphical representation of these results. Standard errors for averages are given in parenesis.


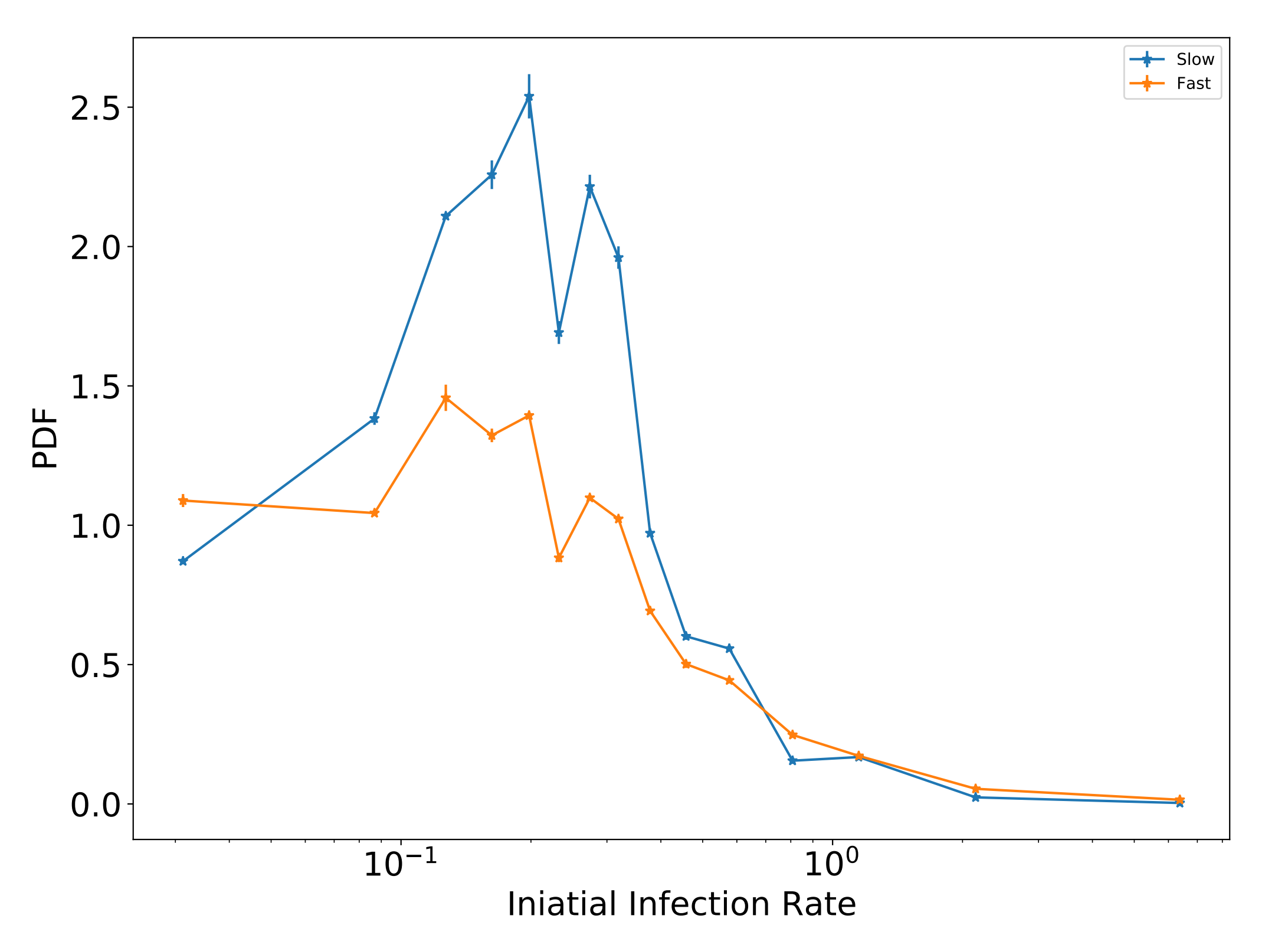


Figure S1. PDF of the initial hourly malware growth rate computed over the first 12h of the outbreak for two populations: slow (with under 50% of their reach in 72h) and fast (with over 50% of their final reach achieved in 72h) spreading. This plot is identical to Fig 1D in the main manuscript but demoinstrates the difference between the distributions on linear scale. Confidence intervals are based on standard errors.


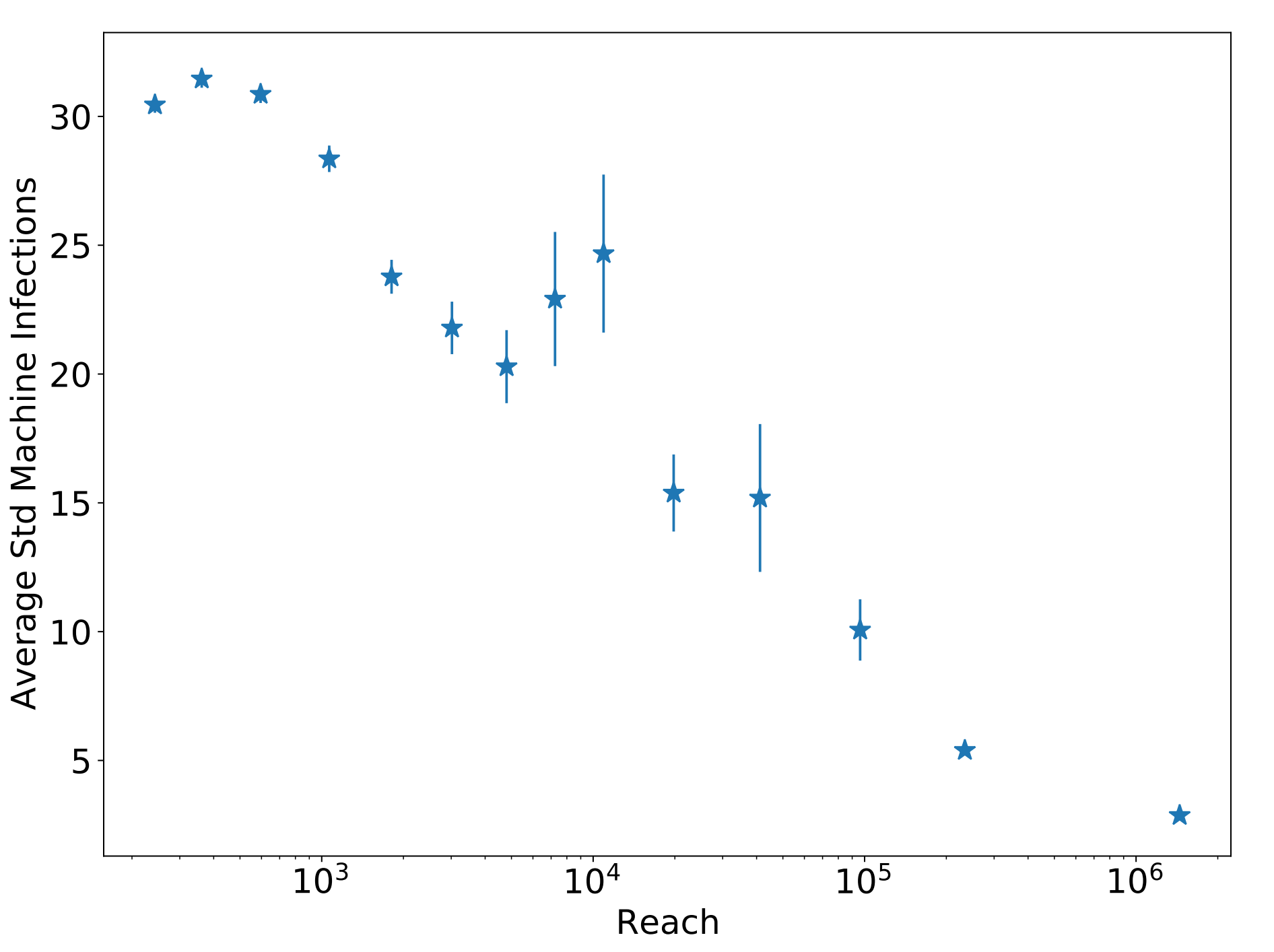


Figure S2: Standard deviation of the susceptibility of the machines infected by a malware during the first 72 hour of its spread, averaged across malware of the same reach as a function of reach. Confidence intervals represent mean standard error for each observation. The figure demonstrates relative homogeneity of the early infected population of the particularly large outbreaks.

# References

1. A. Klaus, S. Yu, D. Plenz, Statistical analyses support power law distributions found in neuronal avalanches. PloS one 6, (2011).

2. J. Alstott, D. P. Bullmore, powerlaw: a Python package for analysis of heavy-tailed distributions. PloS one 9, (2014).
