# Supplementary material for: Initial growth rates of malware epidemics fail to predict their reach
Source: Sci Rep. 2021 Jun 3;11:11750. doi: 10.1038/s41598-021-91321-0 (PMC8175743; doi:10.1038/s41598-021-91321-0)
Supplement: Supplementary file 2 — Supplementary Information 2. [file 41598_2021_91321_MOESM2_ESM.zip › Supp_Mat/READ.ME.pdf]

Stochastic SIR model with varying infectivity.

Main code – Run_Sims.

The attached code contains two types of simulations:

- Fully mixed simulations.
- Network-based simulations.

Both simulations include variable infectivity for each susceptible, and no variability on the probability to infect other susceptibles.

The code in Matlab can be run through a master code called Run_Sims.m, which can run any of the two simulations above.

The code for the fully mixed simulations and the network simulations are respectively:

- Epi_Sim(beta,delta)
- Epi_Sim_Network(beta,delta)

The only two parameters transferred to the code are beta and delta, which are the baseline probability to be infected and the probability of a susceptible to become removed.

The parameters used in the paper are a log scale from 1.e-6 to 1.e-1 for beta, and 1.e-4 to 1.e-1 for delta.

The simulations return:

1. The infected population at each time point – Inf_Pop
2. The cumulative infected population Inf_Cum.
3. Beta_His is the list of beta values of the infected susceptible at the appropriate time point. At each time point, a single event can occur. All are of the length of the number of time steps. The maximal number of time steps is hard-coded, but the simulation stops if there are no infected left.
4. Beta_Keep – the original distribution of beta values used to seed the simulation

Simulation code - Epi_Sim and Epi_Sim_Network

Each of the two simulations uses a tree formalism for the events, and at each time point, a single event is performed. In the current simulations – either infection or the removal of an infected. Following each event, the probabilities are updated in the appropriate tree.

The main structure of the code is as follow:

A) Seeding the trees with the probabilities for the events. The trees contain the total population and are limited $N_{Size} = 2^{Depth-1}$, where Depth here is currently 16. The trees are applied using an array, where position i points to its two descendants are positions 2*I and 2*i+1 (starting from 1). The root of the tree (position 1), is the total probability for an event, which is the sum of its two direct descendants, and so on recursively to the leaves, which are individuals.

B) Seeding N_Init infected individuals (Current N_init=5), and update probability trees.
C) Loop over time points. For each time point:
   a. Decide whether the next event is removal or infection
   b. Choose the individual/vertex to perform the event
   c. Update the probabilities.
   d. The loop ends, when the maximal number of time points ($N_{Steps} = 2 * N_{Size}$)is reached, or the number of infected is zero.

The choice of the individual to infect is through a regular tree traversal, as described in :

Invasion Rate Versus Diversity in Population Dynamics with Catastrophes - A. Melka, N. Dori, and Y. Louzoun - Phys. Rev. Lett. 124, 158301

The initial seeding of probabilities is a constant delta for each individual as supplied in the input to the function, and a variable beta value. Each individual is either assigned a beta value proportional to the beta supplied as input to the function multiplied by a value between 1 and K, distributed with a power law with a slope of 2.5 (using the functions SF_Choice and SF_Choice_Network), or assigned a number of neighbors with the same distribution, each with a probability of beta to infect.

There are no other free parameters in the simulations. If one wants to change the slope, it must be changed in the code in SF_Choice and SF_Choice_Network

When updating the probabilities in the network model, the probability to infect of each of the neighbors of the infected nodes is also reduced, since they have one less susceptible neighbor.

As stated above, while the in-degree distribution is a power law, the out-degree distribution is uniform to avoid variability in the probability of infecting other nodes.
